# Cysteine-113 covalency inspires the development of Pin1 inhibitor

**DOI:** 10.1038/s41392-020-00339-9

**Published:** 2020-10-06

**Authors:** Wenchen Pu, Jiao Li, Yong Peng

**Affiliations:** Laboratory of Molecular Oncology, Frontiers Science Center for Disease-related Molecular Network, State Key Laboratory of Biotherapy and Cancer Center, West China Hospital, Sichuan University, Chengdu, 610064 China

**Keywords:** Molecular medicine, Drug development

Peptidyl-prolyl cis-trans isomerase NIMA-interacting 1 (Pin1) controls the functional switch of phosphoproteins and plays an oncogenic role in human cancer, but the discovery of effective Pin1 inhibitors remains challenging. A recent study by Pinch et al. developed a novel Pin1 inhibitor by endowing molecule with covalent reactivity toward Pin1 cysteine-113,^1^ providing new insights into the design of anticancer Pin1 inhibitors.

The phosphorylation of Serine or Threonine residues preceding a Proline (Ser/Thr-Pro), accounting for more than one-quarter of global phosphorylation sites in human cells, plays a central role in numerous signal transduction and cellular processes. Of all the natural amino acids, proline is unique to adopt either cis or trans conformations due to its imidic peptide bond in backbone. Pin1 is the only known enzyme that specifically catalyzes the cis-trans isomerization of phosphorylated Ser/Thr-Pro (pSer/Thr-Pro) motif, controlling the biological activity, conformation, stability, and subcellular localization of Pin1 substrates.^2^

**Fig. 1:**
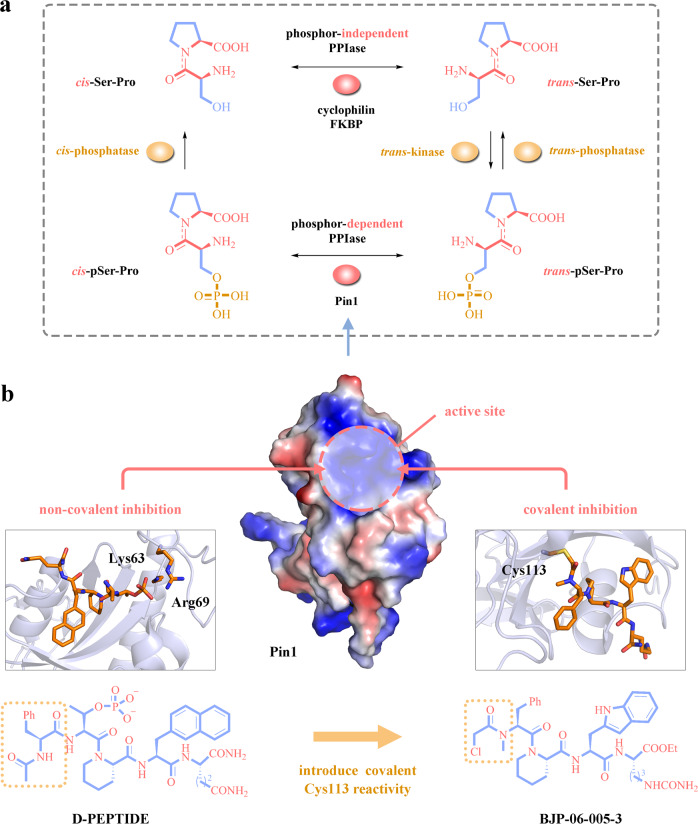
Pin1 is a molecular switch of protein function and an attractive anticancer target available for non-covalent or covalent inhibition. **a** The cis-trans interconversion of unphosphorylated Ser/Thr-Pro motifs is catalyzed by phosphor-independent PPIases, such as cyclophilins and FKBPs. In the presence of trans-specific kinase (trans-kinase), trans-Ser-Pro motif phosphorylated to trans-pSer-Pro, whereas its dephosphorylation process is mediated by trans-specific phosphatase (trans-phosphatase). Pin1 is the only known phosphorylation-dependent PPIase that activates the cis-trans rotation of pSer-Pro motif, and cis-pSer-Pro could convert into cis-Ser-Pro with an aid of cis-specific phosphatase (cis-phosphatase). Thus, Pin1 is a molecular switch of Ser/Thr-Pro segment, leading to a functional diversity or distinctly pathological consequences of Pin1 substrates. **b** The active site of Pin1 PPIase domain is an attractive target for Pin1 inhibition, inducing the discovery of non-covalent and covalent Pin1 inhibitors. Pinch et al. introduce Cys113 covalent reactivity into D-PEPTIDE to obtain BJP-06-005-3 as the first peptidyl covalent Pin1 inhibitor. Covalent Pin1 inhibitor BJP-06-005-3 targets Pin1 Cys113 by forming a carbon–sulfur bond, while non-covalent Pin1 inhibitor D-PEPTIDE interacts with Pin1 Lys63 and Arg69 under an irreversible manner

Pin1, originally identified in 1996 by Lu et al., has a molecular architecture containing a N-terminal WW domain and a C-terminal PPIase domain. The WW domain guides Pin1 close to phosphoproteins via specifically recognizing pSer/Thr-Pro motif, whereas the PPIase domain subsequently catalyzes the conformation change of its substrates. Theoretically, there are four possible models of Ser/Thr-Pro segment considering its phosphorylation status (regulated by kinases and phosphatases) and peptide conformation (regulated by PPIases), leading to a functional diversity or distinctly pathological consequences of given substrates. In this interconversion, Pin1 spatially and temporally targets pSer/Thr-Pro to mediate cis-to-trans or trans-to-cis isomerization, and acts as a molecular switch of protein function (Fig. 1a).^2^

Pin1 dysregulation involves in the pathogenesis of multiple human diseases. Downregulation of Pin1 in brain usually causes Alzheimer’s disease, while Pin1 is frequently overexpressed and/or overactivated in diverse cancers, including leukemia, prostate cancer, and hepatocellular carcinoma (HCC). Emerging evidence has suggested that Pin1 promotes tumor initiation and progression by activating oncogenes. For example, Pin1 isomerizes phosphorylated Thr286-Pro motif of cyclin D1 (an oncogenic cell-cycle regulator) to enhance its nuclear accumulation and stability, triggering cell-cycle progression.^2^ Pin1 also exerts its oncogenic function via inactivating tumor suppressors or growth-inhibitory regulators. XPO5 represses HCC by potentiating the biogenesis of microRNAs, a class of small noncoding RNAs that regulate gene expression by repressing protein translation and generally inhibit cancer development. Pin1 mediates the twist of XPO5 Ser497-Pro to block XPO5 activity, thereby impeding miRNAs biogenesis and promoting HCC development.^3^ These findings make Pin1 an attractive target for cancer therapy and induce the discovery of Pin1-targeted inhibitors.

Since the discovery of juglone as the first Pin1 inhibitor in 1998, diverse Pin1 inhibitors were developed to treat Pin1-associated cancers or probe Pin1 biology. These inhibitors could be classified into non-covalent and covalent Pin1 inhibitors according to their mode of interaction. Non-covalent Pin1 inhibitors, including small-molecule inhibitors (ATRA, API-1, et al.) and peptidyl inhibitors (D-PEPTIDE, et al.), reversibly associate with Pin1 via non-covalent forces such as hydrogen bond and hydrophobic interaction, in which Pin1 lysine-63 (Lys63) and arginine-69 (Arg69) are key residues that recruit inhibitors to approach Pin1. For instance, small-molecule API-1 interacts with Pin1 Lys63 and Arg69 via non-covalent hydrogen bond or π interaction, suppressing HCC via enhancing the expression of mature miRNAs.^4^ Peptidyl non-covalent inhibitors are developed to mimic Pin1 substrate and often have a high Pin1 affinity. The phosphate moiety of peptidyl inhibitor D-PEPTIDE directly contacts to Lys63 and Arg69 and inhibits Pin1 enzymatic activity in vitro with an IC_50_ value of 1 nM, which is one of the most potent Pin1 inhibitors so far (Fig. 1b).^2^

Covalent inhibitors targets Pin1 under an irreversible mechanism by forming covalent bonds, usually with an improved potency and prolonged duration of action. Pin1 cysteine-113 (Cys113) is critical in the action of covalent inhibitors, whereas Cys113 only provides weak interaction (such as π–alkyl interaction and van der Waals force) or even steric hindrance in the presence of non-covalent Pin1 inhibitors.^2,4^ Cys113 is an evolutionally conserved residue in the active site of PPIase domain and has a mercapto group providing adequate nucleophilicity to form covalent bond with strong electrophiles (such as sulfanyl-acetate group or Michael acceptors) within inhibitors. For example, small-molecule KPT-6566’s sulfanyl-acetate group attacks the sulfur atom of Pin1 Cys113 to form a disulfide bond and decrease Pin1 activity, impairing Pin1-dependent cancer phenotypes by downregulating cyclin D1.^5^ However, there are no reports on peptidyl covalent Pin1 inhibitors until the works of Pinch et al.

In a recent study in Nature Chemical Biology, Pinch and his colleagues discovered a novel covalent Pin1 inhibitor. Based on a peptidyl non-covalent Pin1 inhibitor D-PEPTIDE that is associated with Lys63 and Arg69, the authors add an α-chloroacetamide as an electrophile to render D-PEPTIDE with a covalent reactivity toward Pin1 Cys113; this makes BJP-06-005-3 (*Ki*: 15 nM) have an increased Pin1 affinity over D-PEPTIDE (*Ki*: 20 nM). Co-crystal data indicate that BJP-06-005-3 occupies Pin1 Cys113 through a covalent carbon–sulfur bond, giving a structural basis for the interaction between BJP-06-005-3 and Pin1 (Fig. 1b).^1^

To the best of our knowledge, BJP-06-005-3 is the first example of peptidyl covalent Pin1 inhibitor. By replacing the phosphate group of D-PEPTIDE with cell-permeable moiety, BJP-06-005-3 also has a better performance in bioavailability and cellular Pin1 selectivity, showing an attractive potential in cancer treatment. Pancreatic ductal adenocarcinoma (PDAC) is one of the most aggressive solid tumors with rapid growth, hidden symptoms, and early metastasis. 90–95% of PDAC cases harbor *KRAS* mutations, which is difficult to develop inhibitors, spurring efforts to target proteins that facilitate Ras signaling, including Pin1. BJP-06-005-3 triggers cell-cycle arrest through modulating myc-Ras signaling, consistently suppressing PDAC cell viability in a Pin1-dependent manner via inhibiting Pin1 activity and inducing Pin1 degradation.^1^ These results suggest that if endowing a covalent Cys113 reactivity to inhibitors that originally of non-covalent binding capacity toward Lys63 and Arg69, as well as optimizing their bioavailability through chemical modifications, there is a chance to get more powerful anticancer Pin1 inhibitors from the aspect of molecular design.

Indeed, BJP-06-005-3 is a promising tool for exploring Pin1 biology in human cancer, but its characteristics still need to be improved considering its clinical application in cancer therapy. In the future, molecular design of Pin1 inhibitor might be carried out by optimizing BJP-06-005-3’s amide bonds to make it resistant to in vivo metabolic system and more bioavailable, or by introducing covalent Cys113 reactivity on small-molecule non-covalent inhibitors to increase their potency and specificity. Moreover, the bioavailability of inhibitors could be further improved by chemical modifications on the premise of guaranteeing their potency, specificity, and stability. In the view of molecular biology, it’s also interesting to utilize BJP-06-005-3 to dissect the accurate mechanism of Pin1 in PDAC oncogenesis, which remains obscure to date.

In summary, Pinch et al. designed a novel D-PEPTIDE-based covalent Pin1 inhibitor through (1) introducing α-chloroacetamide to quantify Cys113 reactivity, (2) replacing amino acid residues to improve affinity, and (3) modifying N-terminus and C-terminus to increase lipophilicity.^1^ The discovery of BJP-06-005-3 provides a powerful tool for interrogating Pin1 biology and new insights into the design of effective anticancer Pin1 inhibitor.
